# Awareness and Knowledge About Glaucoma in the Western Region of Saudi Arabia: A Population-Based Survey

**DOI:** 10.7759/cureus.47090

**Published:** 2023-10-16

**Authors:** Huda Ahmedhussain, Nooran O Badeeb, Firas Madani, Lujain Z Khawandanh, Essa A Al-Abbas, Osama Badeeb

**Affiliations:** 1 Ophthalmology, Faculty of Medicine, King Abdulaziz University, Jeddah, SAU; 2 Ophthalmology, University of Jeddah, Jeddah, SAU; 3 Internal Medicine, King Abdullah Medical Complex, Jeddah, SAU; 4 Ophthalmology, King Faisal Medical City, Abha, SAU

**Keywords:** population, saudi, knowledge, awareness, glaucoma

## Abstract

Background

Glaucoma is a leading cause of irreversible blindness worldwide. This study aimed to assess the Saudi population’s levels of awareness and knowledge regarding glaucoma risk factors, symptoms, treatment, and outcomes.

Methods

A cross-sectional study was conducted among the Glaucoma Awareness Campaign attendees during the World Glaucoma Week (2015-2016). A structured questionnaire was used, and a knowledge score (0-25) was calculated as the sum of all correct answers. Sociodemographic factors, personal and family history of glaucoma, and the presence of risk factors were investigated and analyzed as factors affecting knowledge.

Results

The study included 1751 participants, with a mean age of 40.23 (SD ±13.86) years; 51.5% were males, 3.7% had glaucoma and 22.6% had a family history of glaucoma. The overall awareness rate was 65.6%, which was moderately higher among females (71.6%), older participants (≥40 years, 69.7%), and highly educated participants (70.6%). Concerning knowledge, 15.4% had fair to good knowledge (score 15-25). Participants with a personal history of glaucoma had relatively greater knowledge regarding glaucoma-specific questions, such as optic nerve damage (p=0.001) and the requirement of lifetime treatment (p<0.001).

Conclusion

Awareness and knowledge about glaucoma are limited among the Saudi population, regardless of socioeconomic class or educational status. Knowledge about glaucoma should be further promoted to enable early screening and prevention.

## Introduction

Glaucoma is the second leading cause of irreversible blindness worldwide [1]. Its global prevalence in 2013 among people aged 40 years and above was estimated at 3.54%, representing almost 64.3 million patients, and is predicted to be rising to 111 million by 2040 [2]. Early detection and treatment are essential to prevent irreversible structural damage to the optic nerve and preserve the patient’s quality of life [3,4].

In Saudi Arabia, the epidemiological data about glaucoma are limited; one population-based study conducted among 785 Saudi citizens aged 60 years and older reported a prevalence of 3.7% for primary open-angle glaucoma (POAG) and 3.9% for primary angle-closure glaucoma (PACG) [5]. In a hospital-based study, glaucoma was reported in 17.7% of patients attending ophthalmology clinics in tertiary care in the western region of Saudi Arabia (30.5% POAG and 24% PACG) [6]. In an ophthalmic university center in the central region, POAG was present in 12.8% and PACG in 46.4% patients [7]. Overall, glaucoma is responsible for 5.8% of visual impairments in Saudi Arabia [8].

Despite the notable progress in glaucoma diagnostics enabling early detection of structural and functional abnormalities, low awareness among the population is responsible for many cases being detected late [3,9,10]. This study assessed the levels of awareness and knowledge about glaucoma among a sample of the Saudi population attending the World Glaucoma Week (WGW) in the western region of Saudi Arabia.

## Materials and methods

A large-scale, cross-sectional survey was conducted during the 2015 and 2016 sessions of the World Glaucoma Week in Jeddah, which took place over the second week of March of each year. The WGW is a joint global initiative by the World Glaucoma Association and the World Glaucoma Patients Association, aiming to raise awareness about glaucoma among the population. Participants were recruited from two main event sites, including the outpatient clinics of a tertiary university center and a large public forum in Jeddah, Saudi Arabia. This study was approved by the Biomedical Ethics Committee at King Abdulaziz University, Jeddah, Saudi Arabia (reference no. 146-14).

A semi-structured questionnaire was distributed to all participants to assess their awareness of glaucoma and their knowledge of the significant risk factors, symptoms, treatment options, and outcomes of glaucoma (25 items). Additionally, the questionnaire explored the primary sources of knowledge, including doctors, the internet, society, TV, and books. The correctness rate was calculated for each item. A knowledge score was calculated as the sum of all correct answers (0-25) and divided into five knowledge levels: very poor (0-4); poor (5-9); average (10-14); fair (15-19) and good (20-25). A glaucoma-specific knowledge score was also calculated, including 13 out of the 25 items considered more specific to glaucoma. These included whether heredity is a risk factor, whether glaucoma affects peripheral vision, and whether the disease could be definitively cured. The questionnaire collected participants’ sociodemographics (age, gender, educational level, and professional status) and clinical data (personal and family history of glaucoma, personal ophthalmological and medical history, glaucoma risk factors such as diabetes, steroid use, and any other eye disease). Sociodemographic and clinical data and knowledge sources were analyzed as factors for awareness and knowledge. The questionnaire was administered either by one of the authors or a trained medical student.

Participants meeting the inclusion criteria were individuals aged 19 and above, encompassing any gender, educational level, and professional status. They were required to have attended the WGW campaigns held in Jeddah, Saudi Arabia, during the 2015 and 2016 sessions. Additionally, participants were expected to have completed the semi-structured questionnaire. Conversely, individuals who were younger than 19 years old and those who exhibited language or cognitive limitations impeding their ability to understand and respond to the questionnaire were excluded from the study.

Statistical methods

Statistical analysis was performed using IBM SPSS Statistics, version 21.0, for Windows (IBM Corp., Armonk, NY). Descriptive statistics were utilized to present participants’ demographic, socioeconomic, and clinical characteristics and the patterns of their answers to the outcome questions, including awareness and various knowledge-related items. The awareness rate was calculated as the percentage of participants who self-declared as being aware of the disease, while correctness rates were calculated as the percentage of participants who answered correctly to the given items. Awareness factors were analyzed by comparing awareness rates between different categories using the chi-square test. Factors of knowledge (general and specific) were analyzed by comparing the mean knowledge scores between different categories using the independent t-test or one-way analysis of variance (ANOVA) by assuming a normal distribution of the two scores. This analysis included only participants who declared being aware of glaucoma. The knowledge level was further compared between glaucoma participants and those without a personal history of glaucoma by analyzing knowledge scores, correctness rates by item, and distribution within different knowledge level categories; independent t-test and chi-square test were used, as appropriate. A p-value <0.05 was considered to reject the null hypothesis.

## Results

Participant characteristics

A total of 1751 participants were recruited (869 in 2015 and 882 in 2016). The following sociodemographic features were observed: 51.5% were males, mean age (±SD) was 40.23 (±13.86) years, 60.9% were highly educated (university plus level), and 64.7% were employed. The clinical characteristics showed that 3.7% declared being diagnosed with glaucoma, 22.6% reported a family history of glaucoma, 20.0% were diabetic, and 21.5% were hypertensive; the prevalence of other ophthalmological disorders ranged from 8.3% for cataract to 38.9% for myopia (Table 1).

**Table 1:** Demographic and clinical characteristics of the participants ^*^Because of missing data, some frequencies do not sum up to the total.

Parameter	Value	Total (N=1751)*	
		N/mean	%/SD
Age, mean ± SD (years)		40.23	13.86
Gender	Male	902	51.5
	Female	849	48.5
Nationality	Saudi	942	53.8
	Non-Saudi	809	46.2
Educational level	Illiterate	23	1.5
	Primary	101	6.7
	Middle	125	8.3
	Secondary	340	22.5
	University +	919	60.9
Professional status	Unemployed	53	3.8
	Housewife	219	15.9
	Employed	891	64.7
	Student	144	10.5
	Retired	70	5.1
Glaucoma	No	1205	68.8
	Yes	65	3.7
	Doesn’t know	478	27.3
Regular ophthalmology follow-up		118	6.7
Family history of glaucoma		395	22.6
Diabetes		350	20.0
Hypertension		377	21.5
Hypermetropia		400	22.8
Myopia		682	38.9
Cataract		146	8.3
Other ophthalmic disease		278	15.9

Awareness and knowledge about glaucoma

Of the participants, 65.6% were aware of glaucoma as a disease. Regarding knowledge, only 29.0% correctly perceived glaucoma as a chronic disease, 51.6% correctly replied that it is linked to the elevation of intraocular pressure (IOP), and 55.4% correctly answered that it affects the optic nerve. Almost half of the patients 51.6% appreciated that glaucoma is a distinct disease than cataract and 50.9% answered that it is different than increased blood pressure. Regarding knowledge about the risk factors, aging was correctly identified by 64.7% of participants, while heredity was identified by only 36.3%, diabetes and hypertension were identified by 53.2 and 14.3% respectively. Among the symptoms, impairment of peripheral vision was correctly identified by 47.7%. The presentation of other symptoms was diverse, with impaired night vision reported by 47.7% patients, followed by headache in 32.6% of cases. Eye pain was experienced by 28.7% patients, while redness of the eye was reported in 24.3% of cases. Halos around lights were observed by 20.2% of patients, and a smaller percentage experienced nausea and vomiting (7.3%) or abdominal pain (2.7%). Regarding treatment, only 21.5%, 28.8% and 44.7% correctly replied that it could be treated with eye drops, laser, or surgery, respectively. Surprisingly, 91.5% thought that glasses can treat glaucoma. Only 27.1% thought that treatment should be continued for life and 27.7% thought that it can be definitively cured.

In terms of the distribution of knowledge levels among the total study population, 18.8% had very poor scores (0-4), 33.1% had poor scores (5-9), 32.7% had average scores (10-14), 14.3% had fair scores(15-19), and only 1.1% had good knowledge scores (20-25).

Concerning the sources of knowledge, society was the most frequent source reported by 35.6% of the participants, followed by the internet (16.5%), doctors (15.5%), and TV (11.3%), and the last one was books as reported by 6.8% participants.

Factors associated with higher awareness and knowledge about glaucoma

Table 2 presents the sociodemographic and clinical factors associated with glaucoma awareness. The awareness rates were moderately higher among older (≥40 years), female, and highly educated participants, as well as those with personal or family history of glaucoma compared to their counterparts, and the differences were statistically significant (p<0.05). Furthermore, the awareness rate was higher among participants who declared having a personal history of diabetes, hypertension, myopia, and other eye diseases, and the differences were statistically significant (p<0.05), except for the case of hypermetropia and cataract (p<0.05).

**Table 2:** Demographic and clinical factors associated with awareness about glaucoma ^*^Statistically significant result, p<0.05

Parameter	Value	Awareness (rate)		
		Freq.	%	p-value
Age (years)	<40	503	61.7	< .001
	≥40	629	69.7	
Gender	Male	533	59.8	< .001
	Female	604	71.6	
Nationality	Saudi	641	69.7	< .001
	Non-Saudi	483	61.0	
Education level	Illiterate	10	45.5	.002*
	Primary	68	67.3	
	Middle	71	57.3	
	Secondary	216	63.9	
	University +	647	70.6	
Professional status	Unemployed	31	58.5	.418
	Housewife	155	70.8	
	Employed	600	67.6	
	Student	97	67.8	
	Retired	41	62.1	
Regular ophthalmology follow-up	No	1031	65.0	.119
	Yes	85	72.0	
Family history of glaucoma	No	653	66.8	.002*
	Yes	297	75.4	
Diabetes history	No	894	64.4	.042*
	Yes	245	70.2	
Hypertension history	No	874	64.1	.015*
	Yes	265	70.9	
Hypermetropia history	No	880	65.7	.904
	Yes	259	65.2	
Myopia history	No	675	63.6	.027*
	Yes	464	68.7	
Cataract history	No	1047	65.7	.657
	Yes	92	63.9	
Other ophthalmic diseases	No	937	64.1	.003*
	Yes	202	73.5	

The general knowledge score was greater among females (p=0.002), glaucomatous individuals (p=0.001), those who had regular ophthalmology follow-ups (p=0.016), those who had a family history of glaucoma (p<0.001), and those who had hypermetropia (p=0.006) compared to their counterparts. The specific knowledge score was higher among glaucoma patients (p=0.002), those who had regular ophthalmology follow-ups (p=0.029), those who had a family history of glaucoma (p<0.001), and those who had hypermetropia (p=0.027) as compared to their counterparts (Table 3).

**Table 3:** Demographic and clinical factors associated with glaucoma knowledge (excluding participants who declared being not aware of glaucoma) ^*^Statistically significant result, p<0.05

Parameter	Value	Knowledge score (N=1139)					
		General			Specific		
		Mean	SD	p-value	Mean	SD	p-value
Age (years)	<40	9.33	4.78	.863	5.88	2.98	.502
	≥40	9.29	4.79		5.79	3.02	
Gender	Male	10.20	4.39	.002*	6.33	2.81	.155
	Female	11.02	4.36		6.56	2.82	
Nationality	Saudi	10.53	4.38	.303	6.49	2.78	.714
	Non-Saudi	10.80	4.43		6.42	2.87	
Education level	Illiterate	9.50	3.98	.875	5.70	2.36	.605
	Primary	10.71	5.14		6.10	3.01	
	Middle	10.55	4.77		6.21	3.12	
	Secondary	10.48	4.40		6.53	2.63	
	University +	10.71	4.14		6.51	2.79	
Professional status	Unemployed	11.48	4.12	.071	6.55	2.69	.346
	Housewife	10.74	4.74		6.48	2.86	
	Employed	10.58	4.28		6.46	2.79	
	Student	11.67	3.47		6.90	2.32	
	Retired	9.68	5.13		5.83	3.22	
Glaucoma	No/don’t know	10.54	4.38	.001*	6.40	2.82	.002*
	Yes	12.69	4.17		7.73	2.54	
Regular ophthalmology follow-up	No	10.52	4.33	.016*	6.39	2.81	.029*
	Yes	11.71	4.93		7.08	2.95	
Family history of glaucoma	No	10.29	4.31	< .001	6.19	2.83	< .001
	Yes	11.85	3.95		7.27	2.54	
Diabetes history	No	10.72	4.42	.195	6.49	2.82	.339
	Yes	10.31	4.31		6.30	2.80	
Hypertension history	No	10.72	4.34	.222	6.53	2.81	.084
	Yes	10.34	4.56		6.19	2.83	
Hypermetropia history	No	10.44	4.41	.006*	6.35	2.84	.027*
	Yes	11.28	4.28		6.79	2.71	
Myopia history	No	10.47	4.28	.134	6.35	2.77	.134
	Yes	10.86	4.55		6.60	2.89	
Cataract history	No	10.58	4.37	.253	6.42	2.80	.270
	Yes	11.13	4.70		6.76	3.01	
Other ophthalmic affection	No	10.62	4.39	.860	6.42	2.82	.491
	Yes	10.68	4.30		6.57	2.82	

Knowledge among glaucoma patients

Glaucoma patients had relatively greater knowledge regarding 5 out of the 13 glaucoma-specific questions, including whether glaucoma is associated with high IOP (80.0% correct answers versus 53.8% in non-glaucoma participants; p=0.001), whether it affects the optic nerve (75.6% versus 60.6%; p=0.044), whether it is different from cataract (75.6% versus 57.3%; p=0.015), whether it could be treated with eye drops (37.8% versus 21.2%; p=0.008), and whether treatment should be continued lifelong (66.7% versus 27.3%; p<0.001). Regarding glaucoma-specific knowledge scores, 24.4% of glaucoma participants had a fair knowledge level in glaucoma-specific knowledge versus 13.0% in non-glaucoma ones (p=0.008). A detailed comparison between glaucoma and non-glaucoma participants, including correctness rates in glaucoma-specific and general questions and knowledge scores, has been presented in Table 4.

**Table 4:** Level of knowledge by item among participants with glaucoma versus non-glaucoma The table presents the percentage of participants who gave the correct answers for each item by group. Q1 was about awareness and is not mentioned here as only participants who reported being aware about glaucoma were included in analysis. ^a^Glaucoma-specific questions *Statistically significant results, p<0.05.

Question			No glaucoma (N=1094)		Glaucoma (N=45)		p-value
			Freq.	%	Freq.	%	
Q2	Is it a chronic disease?		414	37.8	28	62.2	.001*
Q3	Is diabetes a risk factor?		724	66.2	30	66.7	.946
Q4	Is hypertension a risk factor?		143	13.1	6	13.3	.959
Q5^a^	Is heredity a risk factor?		484	44.2	22	48.9	.539
Q6	Is aging a risk factor?		870	79.5	32	71.1	.173
Q7	Are there symptoms?		574	52.5	25	55.6	.684
Q8.1^a^	Does it affect peripheral vision?		626	57.2	30	66.7	.209
Q8.2	Does it cause eye pain?		346	31.6	26	57.8	< .001
Q8.3	Does it affect night vision?		368	33.6	19	42.2	.233
Q8.4	Does it cause nausea?		98	9.0	5	11.1	.622
Q8.5	Does it cause redness of eye?		296	27.1	19	42.2	.026*
Q8.6	Does it cause headache?		412	37.7	18	40.0	.751
Q8.7	Does it provoke halos?		265	24.2	14	31.1	.292
Q8.8	Does it cause abdominal pain?		26	2.4	1	2.2	.947
Q9^a^	Does it associate high intraocular pressure?		589	53.8	36	80.0	.001*
Q10^a^	Is there difference between blood pressure and eye pressure?		616	56.3	29	64.4	.280
Q11^a^	Does it affect optic nerve?		663	60.6	34	75.6	.044*
Q12^a^	Is it different from cataract?		627	57.3	34	75.6	.015*
Q13.1^a^	Could it be treated with glasses?		1020	93.2	29	86.7	.091
Q13.2^a^	Could it be treated with eye drops?		232	21.2	17	37.8	.008*
Q13.3^a^	Could it be treated with surgery?		554	50.6	21	46.7	.601
Q13.4^a^	Could it be treated with laser?		353	32.3	14	31.1	.871
Q14^a^	Could it be definitively cured?		309	28.2	17	37.8	.166
Q15^a^	Should treatment be continued all life long?		299	27.3	30	66.7	< .001
Q16^a^	Does treatment improve vision?		627	57.2	25	55.6	.815
Knowledge score (0-25); mean, SD			10.54	4.38	12.69	4.17	.001*
Specific knowledge score; mean, SD			6.40	2.82	7.73	2.54	.002*
Knowledge level		Very poor (0-4)	110	10.1	1	2.2	.018*
		Poor (5-9)	325	29.7	9	20.0	
		Average (10-14)	451	41.2	18	40.0	
		Fair (15-19)	194	17.7	16	35.6	
		Good (20-25)	14	1.3	1	2..2	
Specific knowledge level		Poor (0-5)	384	35.1	7	15.6	.008*
		Average (6-9)	568	51.9	27	60.0	
		Fair+ (10-13)	142	13.0	11	24.4	

## Discussion

This study highlighted the Saudi population's inadequate awareness and insufficient knowledge about glaucoma; although it showed a 65.5% awareness rate, only 15.4% had adequate or good knowledge about the disease. Participants’ age, gender, and educational level had a moderate impact on awareness, while no notable correlation was found between knowledge and demographic factors. Awareness about glaucoma constitutes a significant public health issue, as it is related to early screening, which is directly related to visual outcomes and disability. Eid et al. reported that almost up to 50% of the patients were legally blind, at least in one eye at the time of their first presentation [6]. This issue probably persists due to a lack of awareness and poor medical knowledge [9].

Several studies have reported low glaucoma awareness, although different questionnaire designs have been used. For example, an Indian study found that 73% of rural residents had heard about the disease; however, only 8.3% were aware of the definition of glaucoma, and 1.89% had knowledge about the disease [10]. Regarding specific aspects of glaucoma, the same study showed that familial predisposition was identified as a risk factor by 21%, while it was correctly identified by 36.3% in our study. Other studies found lower levels of awareness; for example, another Indian population-based study found a 13.5% awareness rate and 7.8% knowledge rate; similar to our findings, this study showed increased awareness in older and female participants and those with a family history of glaucoma, whereas knowledge was not associated with age or gender. However, this study showed a significant association between educational levels and knowledge [11]. An Iranian population-based study reported a 46.6% glaucoma awareness rate among Tehran residents aged above 45 years. Only 19.2% could define the disease correctly, and the knowledge level was higher among females and highly educated participants [12]. Another population-based study from Nepal reported an even lower awareness level (2.43%), and awareness was higher among older and male participants [13]. Although these studies used different designs, their results concorded inadequate awareness and knowledge levels among the general population. This emphasizes the need for more efficient, large-scale communication strategies to increase awareness and improve knowledge, improving early diagnosis and visual outcomes.

Remarkably, the knowledge level among participants with glaucoma was relatively satisfactory; 31.1%-46.7% of glaucoma patients correctly identified the therapeutic methods used in glaucoma (eye drops, laser, or surgery) and 66.7% were aware of the treatment’s lifelong continuousness. Furthermore, eye pain and headache were identified as symptoms of glaucoma by 57.8% and 40.0% of glaucoma patients, respectively, while in Ghana, they were identified by 18% and 31% of POAG patients, respectively. Additionally, 44.4% of glaucoma patients acknowledged that glaucoma might have no symptom, compared to 14% patients in Ghana [14]. A Nigerian study found that 41.7% of the glaucoma patients had a poor perception of the disease, comparable to the relatively low glaucoma-specific knowledge levels reported in the present study [15]. Such observations emphasize eye specialists’ role in educating patients about their condition and highlight the need to design adapted educational material and implemented methods after diagnosis.

In the present study, knowledge sources’ analysis demonstrated knowledge transmission through social methods and medical education. This was indicated by society being the primary knowledge source and higher levels of awareness and knowledge among participants with a family history of glaucoma. This encourages taking advantage of social mechanisms to raise awareness, enhance knowledge about glaucoma, and recruit high-risk individuals for screening, especially patients’ relatives. Comparably, “word of mouth” was the primary source of knowledge (46%) about glaucoma in India, followed by healthcare professionals (33%) and mass media information (21%) [10]. Conversely, a study from Ghana showed that 53% of POAG patients obtained data from their healthcare providers, while a minority (11%) heard about it from patients [14]. In Nepal, mass media was the primary information source (40.3%), followed by hospital staff (13.8%) and friends (11.9%) [13].

One limitation of this study is that it focuses on glaucoma awareness and knowledge in earlier years. Since the study does not cover the current period, it may not accurately reflect the current level of awareness among the population. The awareness and knowledge about glaucoma may have changed over time due to various factors, such as advancements in medical research, public health campaigns, and increased access to information. Therefore, the findings of this study may not be representative of the current level of awareness about glaucoma. Another limitation of this study is that the data on glaucoma awareness and knowledge are self-reported by the participants. Self-reported data may be subject to recall bias or social desirability bias. These limitations highlight the need for further research to address the current level of glaucoma awareness and knowledge among the population under investigation.

## Conclusions

According to our study findings, the Saudi population displayed insufficient awareness and knowledge regarding glaucoma, regardless of the socioeconomic class or educational status. This study provides recommendations for enhancing awareness and knowledge, including utilizing general physicians and healthcare professionals as valuable sources of information to educate the public about glaucoma. By improving patients' understanding, compliance with treatment and follow-up can increase, and awareness can be transmitted to their relatives and friends. Additionally, leveraging social networks as a means of public awareness and health-seeking behavior can be effective, employing innovative strategies to raise awareness and encourage individuals to undergo screening.

## References

[1] Quigley HA, Broman AT (2006). The number of people with glaucoma worldwide in 2010 and 2020. Br J Ophthalmol.

[2] Tham YC, Li X, Wong TY, Quigley HA, Aung T, Cheng CY (2014). Global prevalence of glaucoma and projections of glaucoma burden through 2040: a systematic review and meta-analysis. Ophthalmology.

[3] Tatham AJ, Medeiros FA, Zangwill LM, Weinreb RN (2015). Strategies to improve early diagnosis in glaucoma. Prog Brain Res.

[4] Quaranta L, Riva I, Gerardi C, Oddone F, Floriani I, Konstas AG (2016). Quality of life in glaucoma: a review of the literature. Adv Ther.

[5] Smith SD, Al-Jadaan I, Jabak MH (2003). The prevalance of glaucoma in a population-based sample of elderly Saudis. Invest Ophthalmol Vis Sci.

[6] Eid TM, el-Hawary I, el-Menawy W (2009). Prevalence of glaucoma types and legal blindness from glaucoma in the western region of Saudi Arabia: a hospital-based study. Int Ophthalmol.

[7] Al Obeidan SA, Dewedar A, Osman EA, Mousa A (2011). The profile of glaucoma in a tertiary ophthalmic university center in Riyadh, Saudi Arabia. Saudi J Ophthalmol.

[8] Al-Shaaln FF, Bakrman MA, Ibrahim AM, Aljoudi AS (2011). Prevalence and causes of visual impairment among Saudi adults attending primary health care centers in northern Saudi Arabia. Ann Saudi Med.

[9] Topouzis F, Anastasopoulos E (2007). Glaucoma—the importance of early detection and early treatment. Eur J Ophthalmol.

[10] Rewri P, Kakkar M (2014). Awareness, knowledge, and practice: a survey of glaucoma in north Indian rural residents. Indian J Ophthalmol.

[11] Sathyamangalam RV, Paul PG, George R (2009). Determinants of glaucoma awareness and knowledge in urban Chennai. Indian J Ophthalmol.

[12] Katibeh M, Ziaei H, Panah E (2014). Knowledge and awareness of age related eye diseases: a population-based survey. J Ophthalmic Vis Res.

[13] Thapa SS, Berg RV, Khanal S (2011). Prevalence of visual impairment, cataract surgery and awareness of cataract and glaucoma in Bhaktapur district of Nepal: the Bhaktapur Glaucoma Study. BMC Ophthalmol.

[14] Nkum G, Lartey S, Frimpong C, Micah F, Nkum B (2015). Awareness and knowledge of glaucoma among adult patients at the eye clinic of a teaching hospital. Ghana Med J.

[15] Mbadugha CA, Onakoya AO (2014). The awareness, perceptions and experiences of primary open angle glaucoma patients in Lagos Nigeria. Sci Rep.

